# Targeted genome-wide SNP genotyping in feral horses using non-invasive fecal swabs

**DOI:** 10.1007/s12686-022-01259-2

**Published:** 2022-03-16

**Authors:** Stefan Gavriliuc, Salman Reza, Chanwoori Jeong, Fitsum Getachew, Philip D. McLoughlin, Jocelyn Poissant

**Affiliations:** 1 Department of Ecosystem and Public Health, University of Calgary, 3280 Hospital Drive, Calgary, AB T2N 4Z6 Canada; 2 Faculty of Veterinary Medicine, University of Calgary, Calgary, AB T2N 4Z6 Canada; 3 Department of Biology, University of Saskatchewan, 112 Science Place, Saskatoon, SK S7N 5E2 Canada

**Keywords:** Amplicon, Conservation genomics, Equid, Feces, Targeted sequencing, Wildlife

## Abstract

The development of high-throughput sequencing has prompted a transition in wildlife genetics from using microsatellites toward sets of single nucleotide polymorphisms (SNPs). However, genotyping large numbers of targeted SNPs using non-invasive samples remains challenging due to relatively large DNA input requirements. Recently, target enrichment has emerged as a promising approach requiring little template DNA. We assessed the efficacy of Tecan Genomics’ Allegro Targeted Genotyping (ATG) for generating genome-wide SNP data in feral horses using DNA isolated from fecal swabs. Total and host-specific DNA were quantified for 989 samples collected as part of a long-term individual-based study of feral horses on Sable Island, Nova Scotia, Canada, using dsDNA fluorescence and a host-specific qPCR assay, respectively. Forty-eight samples representing 44 individuals containing at least 10 ng of host DNA (ATG’s recommended minimum input) were genotyped using a custom multiplex panel targeting 279 SNPs. Genotyping accuracy and consistency were assessed by contrasting ATG genotypes with those obtained from the same individuals with SNP microarrays, and from multiple samples from the same horse, respectively. 62% of swabs yielded the minimum recommended amount of host DNA for ATG. Ignoring samples that failed to amplify, ATG recovered an average of 88.8% targeted sites per sample, while genotype concordance between ATG and SNP microarrays was 98.5%. The repeatability of genotypes from the same individual approached unity with an average of 99.9%. This study demonstrates the suitability of ATG for genome-wide, non-invasive targeted SNP genotyping, and will facilitate further ecological and conservation genetics research in equids and related species.

**Supplementary Information:**

The online version contains supplementary material available at 10.1007/s12686-022-01259-2.

## Introduction

Molecular genetic markers provide invaluable information for population genetics and evolutionary ecology research, and for characterising, managing and conserving biodiversity (Andrews et al. 2018; Carroll et al. 2018; Hohenlohe et al. 2021). The burgeoning fields of genomics and next-generation sequencing (NGS) have led to increasingly large amounts of DNA sequence data being generated for natural populations (Allendorf 2017; Andrews et al. 2018; Hohenlohe et al. 2021), though the potential of large-scale genomics has yet to be realized in wildlife conservation genetics (Shafer et al. 2015; Andrews et al. 2018). One of the main impediments to applying genomic tools in wildlife is the regular need for collecting minimally or non-invasive samples, which often contain DNA that is highly fragmented, present in low quantities, or contaminated by exogenous DNA from the environment or digesta (Andrews et al. 2018; Carroll et al. 2018). Among non-invasive sampling sources, feces are often readily available in many natural systems and are particularly suitable for sampling when individuals are elusive, or where invasive sampling is either prohibited or dangerous (Carroll et al. 2018; King et al. 2018; White et al. 2019). However, genomic analyses are currently limited for fecal samples due to a lack of flexible and economical technologies.

Numerous approaches have been developed to genotype sets of molecular markers that vary in genomic coverage, cost, and level of throughput. Microsatellites have been the cornerstone of wildlife genetics over the past decades due to their ease of development and genotyping (Selkoe and Toonen 2006). While still widely used (Ferreira et al. 2018; Mengüllüoğlu et al. 2019), microsatellites are rapidly being replaced by single nucleotide polymorphisms (SNPs) which are more abundant across the genome and more amenable to automated high-throughput genotyping. Large SNP panels have been used extensively in humans and agricultural species by way of microarrays (Kim and Misra 2007; Gurgul et al. 2014). However, the prohibitive development cost of dense SNP microarrays has limited their use in wildlife to a handful of species (e.g., Hagen et al. 2013; Malenfant et al. 2015; Kim et al. 2018). SNP microarrays are also ill-suited for non-invasive samples because they typically require large amounts of DNA (Carroll et al. 2018). Genotyping by sequencing (GBS) approaches have circumvented issues with the large development cost of SNP microarrays, but the random distribution of enzymatic cut sites prevents targeting specific loci (Barchi et al., 2019, Scaglione et al., 2019), and most GBS techniques also require amounts of template DNA exceeding what can typically be obtained from non-invasive samples such as feces. Among contemporary genotyping methods, there has been difficulty in achieving a balance between high coverage across the genome while maintaining low development costs and flexibility over target loci, especially for samples collected non-invasively containing low amounts of DNA.

Alongside the development of SNP microarrays and GBS techniques relying on restriction enzymes, target enrichment methods have emerged as viable alternatives for obtaining genome-wide genotype data (Kozarewa et al. 2015; Meek and Larsen 2019). Target enrichment can broadly be classified into methods of sequence capture, where probes are designed as baits for capturing DNA, or through PCR where probes are designed for sequence amplification (Meek and Larsen 2019). By operating on a subset of genetic loci, these methods can provide higher coverage at loci of interest at reduced costs per sample and are often suitable for samples with low DNA content. As such, target enrichment has successfully been used to genotype samples obtained non-invasively, and may be poised to transition the field of wildlife genetics to genomics (Meek and Larsen 2019). A novel targeted sequencing approach by Tecan Genomics (Redwood City, United States) has shown promise for providing genotype data that is cost-effective, high-quality, and high-throughput (Allegro Targeted Genotyping, hereafter “ATG”). Through single primer enrichment technology (SPET), ATG can perform multiplex enrichment of 1 k–100 k+ target loci in a single reaction with a minimal recommended input of 10 ng of DNA. The use of single primers reduces the occurrence of primer dimers and the high specificity for target sites further increases reproducibility between experiments. This approach is also flexible, permitting rapid custom assay design for relatively small number of samples (currently 192), especially when compared to microarray panels which are generally immutable. ATG initially saw uses in human medicine (Scolnick et al. 2015; Nairismägi et al. 2016; Saber et al. 2017), then in plant genetics including black poplar and maize (Scaglione et al. 2019), tomato and eggplant (Barchi et al. 2019), palm oil (Herrero et al. 2020) and endangered plants in the Canary Islands (Gramazio et al. 2020), but its use in wildlife remains limited.

In large mammals, feces are often the most readily available sample tissue for genotyping (King et al. 2018; White et al. 2019). Notably, hindgut fermenters such as equids consume large quantities of forage and accumulate large deposits of epithelial cells in their feces through intestinal abrasion, creating a highly mucosal surface for sampling (Costa et al. 2016; King et al. 2018). Though previous work has shown that fecal swabs sampled from fresh elephant dung contain sufficient DNA for genome-wide genotyping (Bourgeois et al. 2019), it is unclear whether ATG may offer a viable option for generating genomics data from fecal samples in such systems given its low DNA input requirement.

In this study, we investigated the potential of ATG to yield high-quality genotypes from fecal swabs collected from fresh feces in feral horses using samples collected as part of a long-term individual-based population study on Sable Island, Nova Scotia, Canada. First, we quantified the amount of host DNA present in fecal swabs using a qPCR assay to determine if samples provided the recommended DNA input for ATG. Since foal feces are typically smaller than feces from older individuals and also appear to have a thinner layer of mucus on them, we also tested if fecal swabs for foals generally yielded less DNA than those collected from older individuals. After determining that most samples contained sufficient DNA for ATG, we developed a pilot panel of 279 SNPs shown to be polymorphic in the study population based on earlier genotyping using commercial equine SNP microarrays and sample types yielding greater amounts of DNA than fecal swabs (hair roots, saliva, and muscle biopsy). Forty-eight fecal swabs collected from 44 individuals previously genotyped using equine microarrays were then genotyped to evaluate congruence between genotypes generated by different technologies, and consistency of genotypes across repeat samples from the same individual.

## Materials and methods

### Study population

**Fig. 1:**
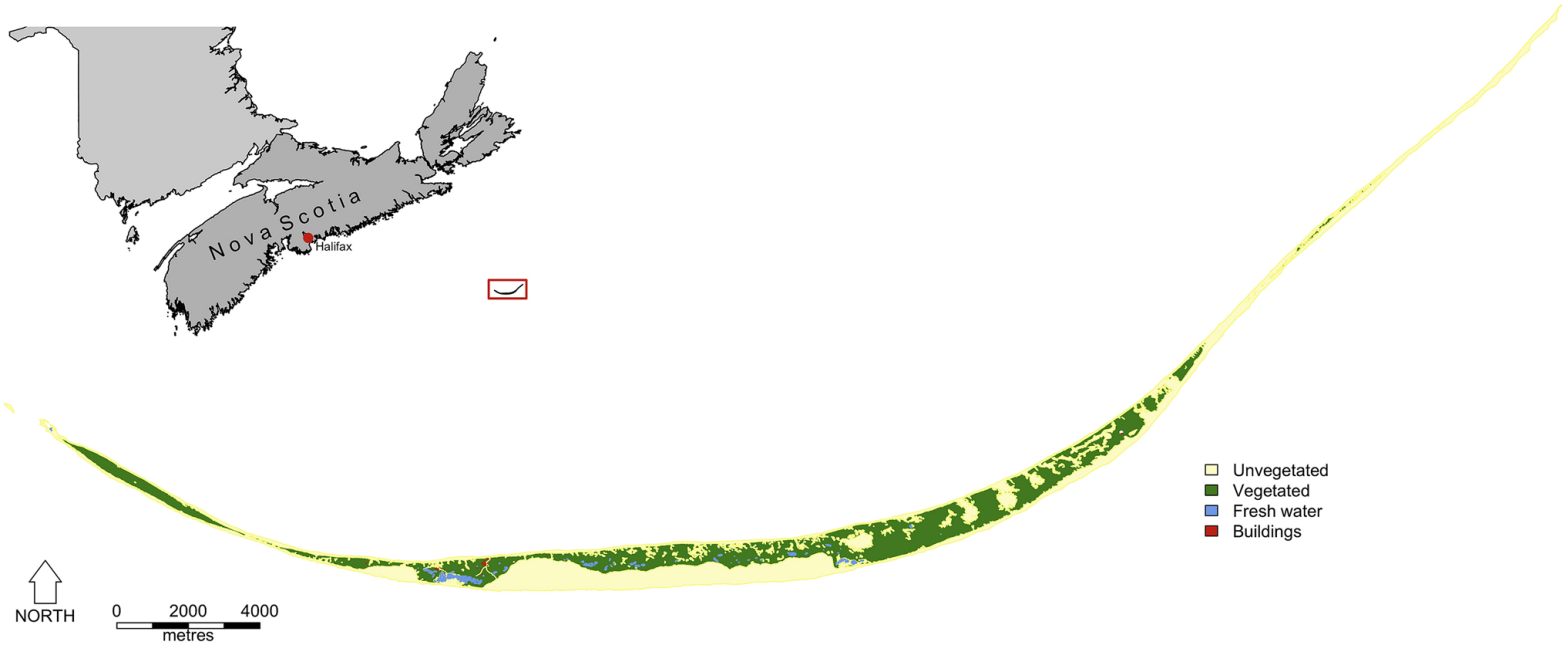
Map of Sable Island National Park Reserve, Nova Scotia, Canada, showing its position relative to the mainland and predominant land cover types (reproduced from Gold et al. 2019)

Sable Island National Park Reserve (43° 55′ N, 60° 00′ W) is an approximately 49-km long and 1.25-km wide sand bar located 275 km southeast of Halifax, Nova Scotia, Canada (Fig. 1). Horses were introduced to the island in the eighteenth century, and have since persisted in a feral state (Christie 1995). Each year since 2007, the horses have been censused during the mid- to late-breeding season (July–September) as part of a long-term individual-based population study (see e.g., Gold et al. 2019; Regan et al. 2020). The island was divided into 7 sections for population monitoring purposes and daily ground surveys were conducted in one of the sections, resulting in a complete coverage of the island during the course of a week (weather permitting). Observers approached bands on foot and recorded horse age (foal, yearling, 2–3 years old, or older), sex, presence of distinct morphological features (e.g., body and facial markings, scars), and composition of social groups. Location was recorded to within 5 m of each individual/group using a hand-held global positioning system (GPS). Photographs of each horse were taken to add to a comprehensive population directory and allow subsequent identification.

### SNP microarray genotyping

Information on SNP positions and allele frequencies in our study population were obtained using various commercial equine SNP microarrays. For these analyses, DNA was obtained from mane and tail hair pulled from live individuals in 2011 and 2012, saliva swabbed from dropped foliage in 2014, and a muscle biopsy obtained from a recently deceased animal. DNA was extracted from hair roots using Qiagen’s User-Developed Isolation of genomic DNA from nails and hair using the QIAamp DNA Mini Kit Protocol (QA05 Jul-10) and the QIAamp DNA Micro Kit, from saliva using DNA Genotek’s protocol, and from muscle tissue using Qiagen’s DNeasy Blood & Tissue Kit following the manufacturer’s protocol. DNA was quantified using 2 µl of template DNA using a Qubit 3 fluorometer and a Broad Range Assay Kit (Thermo Fisher Scientific) according to manufacturer protocols. DNA was then air-dried and shipped to Geneseek/Neogen (Lincoln, United States) for microarray genotyping. A total of 272 samples representing 259 individuals were genotyped. One hundred and eighteen samples were genotyped using the Equine GGP65 array (65,157 SNPs), 120 samples were genotyped using the Equine GGP65 Plus array (71,947 SNPs), and 34 samples were genotyped using the Affymetrix Axiom Equine array (670,796 SNPs).

### Targeted SNP sequencing panel design

To design the panel for ATG, 300 SNPs present on the Illumina Equine GGP65 Plus array and shown to be polymorphic in the Sable Island population were selected. SNPs with minor allele frequency (MAF) > 0.30 and exhibiting limited linkage disequilibrium as determined by the PLINK-*indep-pairwise* command with a window size of 50, a step size of 5 and a variance inflation factor of 0.5 (Purcell et al. 2007) were selected. An assay was then developed by Tecan Genomics (Redwood City, United States) covering 279 of the 300 originally submitted SNPs (Online Table 1). In this assay, 237 targets were covered by 2 probes while 42 were covered by a single probe.

### Fecal DNA samples collection

Horse DNA was collected by swabbing the mucus layer surrounding freshly deposited feces using a polyester swab attached to a 5 mL vial (SIMPORT T307-5A). Vials were preloaded with 400 µl of Aquastool™ solution (MultiTarget Pharmaceuticals) and kept in insulated bags containing icepacks after collection in the field and transferred to – 20 °C when returning to the laboratory on the same day. Samples were transported by air to the mainland (frozen) at the end of each field season and archived at − 80 °C until DNA extraction.

### Fecal DNA extraction

DNA was isolated using a modified version of the Aquastool™ Solution recommended protocol (MultiTarget Pharmaceuticals). First, thawed swab vials were vortexed at full speed for 1 min, and 200 µl of homogenized solution was transferred to a 1.5 ml microfuge tube. Samples were then incubated at room temperature for 15 min, vortexed for 60 s, and centrifuged at full speed on a microcentrifuge (14,000 rpm) for 5 min to pellet debris. The clear supernatant (~ 200 µl) was transferred to a 1.5 ml tube pre-loaded with 160 µl of isopropanol and vortexed for 10 s. Tubes were then centrifuged at full speed for 5 min, and the supernatant removed and discarded. DNA pellets were then rinsed twice with 70% ethanol before being air dried and resuspended in 60 µl of molecular grade water. Once DNA pellets dissolved, samples were centrifuged at full speed for 5 min to pellet contaminants. Clear supernatant containing DNA were transferred to new cryotubes, and archived at − 80 °C.

### Fecal DNA quantification

We quantified total (host + exogenous) DNA concentration in samples using 2 µl of template DNA and a Qubit 4 or BioTek Synergy LX Multi-Mode Microplate Reader with a Qubit or Quant-It dsDNA High-Sensitivity or Broad Range Assay Kit (Thermo Fisher Scientific) according to manufacturer protocols. To assess how much of the total DNA was attributable to host, we applied a qPCR approach targeting the single copy nuclear F2 gene using equine-specific primers known to be effective across horse breeds (Forward: 5′-GCCAGCAGGCTGAGAACG-3′, Reverse: 5′-TGGTGCAGTTGATTCTGGAATAGGAAATTT-3′; Floren et al. 2015) and horse DNA extracted from muscle tissue as a standard (10× dilution series: 20 ng/μl–0.0002 ng/μl). Samples, standards, and negative controls were run in duplicate using a Bio-Rad CFX96 qPCR System, with each reaction containing 2 μl of template, 10.0 μl of 2xSEnsiFAST SYBR MIX, 0.8 μl of 10 μM forward primer, 0.8 μl of 10 μM reverse primer, and 6.4 μl molecular grade water. Thermocycling conditions consisted of 95 °C for 3 min (for polymerase activation) followed by 40 amplification cycles (95 °C for 5 s, 60 °C for 10 s, and 72 °C for 10 s).

### Testing for effect of age on amount of host DNA

We tested whether feces from foals generally had a different amount and proportion of horse DNA compared to feces from older individuals using t-tests. Values were log-transformed [log (*X* + 1)] prior to analysis to approximate normal distributions.

### Library preparation and sequencing

Forty-eight fecal swab samples containing at least 10 ng of host DNA collected from 44 individuals that had previously been successfully genotyped on a commercial SNP array were selected for library preparation and sequencing. Among the 44 individuals sampled, two had two replicate samples and one had three replicate samples obtained on different days. Of the 48 samples, 19 had been genotyped using the Affymetrix 670 k Equine array, 16 had been genotyped using the Illumina Equine GGP65 array, and 13 had been genotyped using the Illumina Equine GGP65Plus array. Library preparation followed the ATG Kit protocol with 10 ng (in 5 μl of molecular grade water) of horse DNA as input. Since DNA concentration of extracts was generally too low for direct inclusion in the library preparation workflow, 5 μl aliquots containing 10 ng of DNA were generated by drying 10 ng of DNA using a SpeedVac Concentrator followed by resuspension in 5 µl of molecular grade water. Libraries were quantified using the PerFeCTa NGS Quantification Kit for Illumina (Quantabio, 95,154) and fragment size analysis with a TapeStation system (Agilent) following the manufacturer’s recommended protocols. Sequencing of a 10 nM library was done on a MiSeq sequencer using the MiSeq V2 (300-cycles) micro kit (Catalog # MS-103-1002).

### Bioinformatics

First, *Trim Galore!* version 0.6.2 (Martin 2011) was used to remove the first 40 base pairs of all forward reads to eliminate synthetic probes and adaptor sequences. To maximize the number of reads retained for downstream analysis, we increased the s*tringency* parameter, denoting the number of overlaps with adaptor sequences required to trim a sequence, from the default value of 1 to 7. The *length* parameter, specifying the minimum threshold in base pairs for retaining a sequence, was set to 15. Remaining reads were aligned to the EquCab2.0 equine reference genome (Wade et al. 2009) using *Bowtie2* version 2.4.2 with the *very-sensitive-local* alignment option enabled (Langmead and Salzberg 2012). Individual.bam files were merged prior to variant calling using the *merge* command from *samtools* version 1.11. To generate a multi-sample variant pileup file, *mpileup* from *bcftools* version 1.11 (Li 2011) was used with the maximum depth set to 10 million reads, the *skip-indels* option enabled, the *-a* parameter set to output the depth per site per sample, and a targets file specifying the chromosome, position, reference and alternate alleles of the 279 targeted sites as determined from the genotypes derived from the Illumina and Affymetrix microarrays. Variants were called using *bcftools call* with: the *-v* parameter enabled to output variant sites only, the *-f* parameter enabled to output genotype quality (GQ) scores, the *-t* parameter with the targets file, and the *-m* parameter enabled for multiallelic calling. Loci that were invariant and genotypes with GQ scores less than 20 were excluded.

### Concordance between genotyping methods and repeat samples

Concordance between genotypes obtained using ATG and commercial microarrays was computed as the proportion of matching alleles (either 0, 0.5, or 1) while ignoring missing genotypes. The consistency of genotypes obtained using ATG was assessed for the three individuals who had multiple samples using the same approach. One individual had three repeat samples and the consistency between genotypes was computed as pairwise concordances using the above scheme.

## Results

### DNA recovered from fecal swabs

**Fig. 2:**
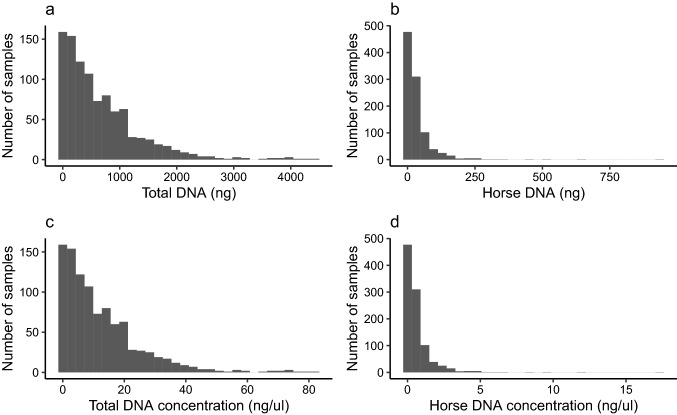
Histograms of the amount (ng) and concentration (ng/ul) of total DNA (**A**, **C**) and host DNA (**B**, **D**) isolated from 989 swab samples collected from freshly voided horse feces on Sable Island, Nova Scotia, Canada. Total and host DNA were quantified using fluorescence and a host-specific qPCR assay, respectively

For the 989 fecal swabs studied, total DNA (host + exogenous) ranged from 1.4 ng to 4428 ng, with a median of 467 ng (Fig. 2A). Host DNA, quantified using a qPCR assay, ranged from 0 to 935 ng with a median of 17 ng (Fig. 2B). Median concentrations of total and host DNA were 8.7 ng/μl and 0.3 ng/μl, respectively (Fig. 2C, D). Approximately 62% of swabs yielded 10 ng of host DNA or more, the minimal input recommended for the ATG kit (Fig. 2B). The percentage of host DNA in samples ranged from 0 to 135% (values > 100% are expected in the presence of measurement error) with a median of 5%.

### Amount and proportion of host DNA in feces of foals versus non-foals

**Fig. 3:**
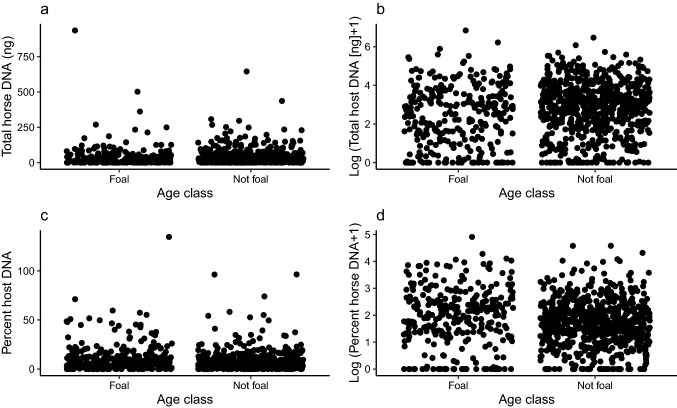
Distribution of total host DNA (**A**), log-transformed total host DNA (**B**), percent host DNA (**C**) and log-transformed percent host DNA (**D**) isolated from fecal swabs collected for foals and older individuals in the population of free-living horses from Sable Island, Nova Scotia, Canada. A total of 989 fecal swabs were collected. Total host DNA and the percentage of DNA attributable to hosts were inferred from separate estimates of host and total (host + exogeneous) DNA obtained using a host-specific qPCR assay and non-specific fluorescence assay, respectively

Overall, 53.2% and 66.6% of swab samples from foals and older individuals yielded more than 10 ng of DNA, respectively. The median amounts of total DNA in swabs from foals and older individuals were 11.8 ng and 19.0 ng, respectively (Fig. 3A), and the difference was significant (log-transformed data, p = 0.0002, Fig. 3B). The median proportions of host DNA in swabs from foals and older individuals were 0.07 and 0.05, respectively (Fig. 3C), and this difference was also significant (log-transformed data, p = 0.0005, Fig. 3D).

### Sequencing and genotyping

**Fig. 4:**
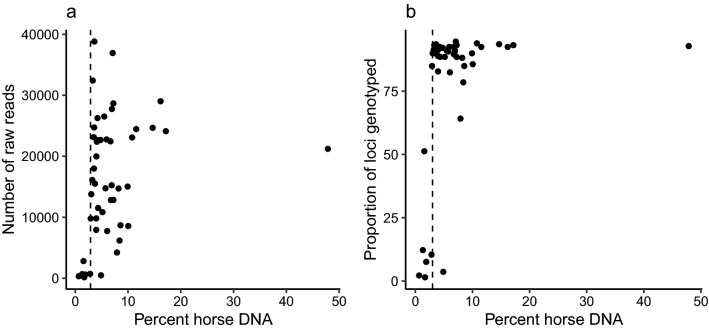
Relationships between the proportion of DNA attributed to the host in a DNA extract and (**A**) the number of raw reads generated and (**B**) the proportion of loci genotyped using an Allegro Targeted Genotyping (ATG) kit targeting 279 SNPs. Fecal swab samples were obtained from 48 freshly voided feces from 44 unique individuals from the free-living population of horses on Sable Island, Nova Scotia, Canada. The proportion of DNA attributable to hosts was inferred from separate estimates of total and host DNA obtained using fluorescence and a host-specific qPCR assay, respectively

**Fig. 5:**
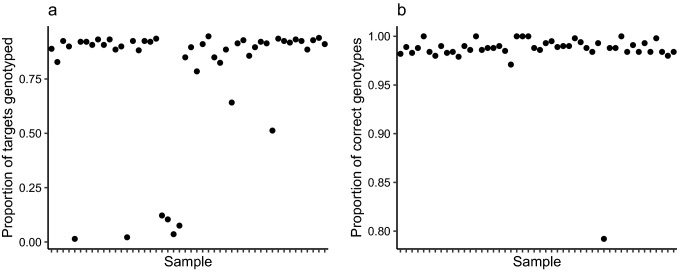
Summary of Allegro Targeted Genotyping for 279 targets for 48 fecal swabs collected from feral horses on Sable Island, Nova Scotia, Canada. Presented are **A** the percent of targets genotyped and **B** the concordance (percent agreement) of genotypes with those from SNP microarrays

The raw number of sequence reads generated for each sample ranged between 154 and 38,817 with a mean ± 1 standard deviation (SD) of 16,345 ± 10,113 (Online Table 2). Six samples had distinctively low numbers of raw reads (< 750, Fig. 4A), suggesting library preparation failure for these samples. All of these were characterised by a relatively low proportion of host DNA, typically < 3% (Fig. 4B). In contrast, library preparation was successful for all but one sample containing > 3% of host DNA (Fig. 4A). An average of 16,010 ± 9906 of raw reads per sample were retained after trimming primers using *TrimGalore!,* and an average of 14,713 ± 9285 reads were successfully aligned to the EquCab2.0 reference genome (Online Resource 3). Prior to variant calling, an average of 12,462 ± 7938 reads were assigned across the 279 targeted sites, and an average of 12,273 ± 7811 reads were retained after variant calling. The number of reads assigned to a target site ranged from 0 to 391 with an average of 47 ± 22 reads per target per sample (Online Resource 4). Out of the 279 targeted loci, one triallelic and 11 invariant sites were identified and removed during the variant calling step. The average number of targets that were assigned a genotype was 248 ± 22 per sample when excluding the 6 samples that failed to amplify and 220 ± 78 when including them (Fig. 5A; Online Resource 4).

### Concordance between genotyping methods and repeat samples

The concordance between genotypes generated using ATG and various commercial equine microarrays ranged from 79.2 to 100% across samples for an average of 98.5% when ignoring triallelic and invariant ATG sites and sites without a genotype (Fig. 5B). Samples that had < 3% horse DNA generally did not amplify (Fig. 4A), providing only several hundred sequence reads and a low proportion of genotyped loci (Fig. 4B). Among the genotypes that did not match between genotyping technologies, 88% were homozygous in the ATG assay, suggesting the presence of null alleles. Lastly, repeat samples from the same individuals provided highly consistent results, with genotype concordances ranging from 99.5 to 100% with an average of 99.9%.

## Discussion

In most species, generating genotypes for large numbers of targeted SNPs remains challenging due to relatively large DNA input requirements and assay development costs. The present study aimed to determine if ATG could be used to generate genome-wide SNP data in feral horses using non-invasive fecal swabs. A relatively large proportion of fecal swabs provided sufficient host DNA for ATG, and most samples submitted to ATG yielded accurate genotypes at nearly all of the targeted SNP loci. Our approach has the capacity to facilitate and broaden ecological and conservation genetics research in horses and other species.

Approximately 62% of swabs analysed yielded sufficient host DNA for ATG (10 ng). Fecal samples have been used extensively in wildlife genetics for amplifying microsatellites (Kierepka et al. 2016; Ferreira et al. 2018; Zhang et al. 2018; Latorre-Cardenas et al. 2020), including feral horses (King et al. 2018, 2021; Schoenecker et al. 2021), but microsatellite genotyping generally does not require the quantification and standardization of host DNA. As a consequence, the amounts of host DNA isolated from fecal samples and whether these meet input requirements of various SNP genotyping technologies are usually unknown. In the case of ATG, *a priori* standardization of DNA inputs is also highly desirable because samples are pooled early during library preparation. Given that recommended inputs for commercial kits are typically conservative to ensure high genotyping success, it is possible that using less than 10 ng of input DNA per sample could still yield valuable data, thus further increasing the proportion of swabs that can be analysed. Alternatively, processing of samples could be changed to collect more mucus than done using a swab, such as drying large amounts of feces and collecting dried mucus from the surface (King et al. 2018).

Quantification of total and host-specific DNA revealed that foal feces yielded less total horse DNA than feces from older horses, but greater proportions of horse DNA relative to contaminants. While the former result aligns with our prediction based on the fact that foal feces are typically smaller and appear to be coated with less mucus than feces from older individuals, the latter was unexpected. Given that host DNA arises in part from intestinal cells released due to abrasion, we expected that the shorter digestive tracts of foals and typically smoother feces because of milk consumption would have resulted in less abrasion and thus lower proportions of horse DNA. To our knowledge, no studies have established a relationship between age and the amount or concentration of host DNA on feces of horses.

The proportion of horse DNA had a large effect on the success of amplification in the ATG assay. Specifically, the few samples for which the assay failed had low proportions of host DNA, typically < 3%. This result was most likely caused by the high amount of non-horse DNA overwhelming kit reagents. Possible solutions to this problem include adding less than the recommended 10 ng of host DNA or using more ATG reagents for problematic samples. Alternatively, the ratio of host DNA in a sample could be increased through methylation-based enrichment (Chiou and Bergey 2018).

Excluding samples that did not amplify, reads assigned to target sites in ATG showed relatively even distribution across samples, and a large proportion of the targeted loci were successfully assigned a genotype per sample. Furthermore, when a target site was assigned a genotype, the genotype identified by ATG displayed high concordance with those generated using commercial equine SNP microarrays. One sample had a comparatively low concordance (79.2%) and is likely attributable to a sample being misidentified as belonging to a different horse during sample collection in the field or samples being mixed up during lab processing. While not addressed specifically in our study, it is likely that genotyping success and accuracy could be increased further by increasing sequencing depth. Eleven loci were invariant when genotyped with ATG, and the large majority of genotyping inconsistencies between genotyping technologies at remaining SNPs (88%) were homozygotes in the ATG assay but heterozygous in the DNA microarray data. These indicate that ATG assays are susceptible to null alleles, which arise from secondary polymorphisms at sites that interfere with genotyping or amplification at the site of interest, such as primer binding regions (Carlson et al. 2006). This finding highlights the need to check for null alleles during future quality control of ATG assays.

While additional research will be required to determine if the method is applicable in other study systems, we anticipate that ATG will work for other species where feces yield high amounts and proportions of host DNA. In particular, ATG should be applicable to other equids, as well as most ungulates for which fecal pellets have been extensively used for microsatellite genotyping (e.g., Brinkman et al. 2011; Poole et al. 2011; Deakin et al. 2020; De et al. 2021), with the caveat of requiring knowledge on SNP loci and flanking sequences. Furthermore, while our study focused on freshly passed feces, it is possible that ATG can be used for fecal samples which have been deposited and left at ambient conditions for a considerable amount of time. In particular, King et al. (2018) showed that mucus from horse feces left in the field for up to two months can yield reliable microsatellite genotypes (King et al. 2018). If successful, such an approach would be highly valuable in many landscape and conservation genomics studies where samples do not have to be linked to a known individual.

Overall, our study shows that, given sufficient host DNA, the ATG assay can recover the genotypes of pre-specified loci with comparable accuracy to SNP microarrays. Applicability of ATG to DNA isolated from fecal swabs as well as other types of non-invasive and invasive samples opens new avenues for genomics studies in horses and other species. In particular, the flexibility of ATG with respect to the position and density of targeted loci, combined with a relatively small minimum order size, should facilitate ecological and conservation genomics research in non-model species.

## Supplementary Information

Below is the link to the electronic supplementary material.

Supplementary file1 (XLTX 18 kb)

Supplementary file2 (PDF 31 kb)

Supplementary file3 (PDF 504 kb)

Supplementary file4 (PDF 676 kb)

## Data Availability

Raw sequence reads are available on Github on Dryad at https://doi.org/10.5061/dryad.0vt4b8h1n.

## Code availability

The code for calling variants is available on Github at “sgavril/Nugen-Pilot-Analysis/”.
