# Supplementary material for: Targeted genome-wide SNP genotyping in feral horses using non-invasive fecal swabs
Source: Conserv Genet Resour. 2022 Mar 16;14(2):203–13. doi: 10.1007/s12686-022-01259-2 (PMC9162989; doi:10.1007/s12686-022-01259-2)
Supplement: Supplementary file 2 — Supplementary file2 (PDF 31 kb) [file 12686_2022_1259_MOESM2_ESM.pdf]

Table 2. Summary table obtained from bioinformatic processing using sequence data generated from the Allegro targeted genotyping kit. Fecal swabs from 44 unique individuals were obtained and enriched for 279 target sites. Detailed are the number of raw reads generated, reads retained during bioinformatic processing, the number of target loci successfully genotyped, and concordance (percent agreement) with SNP chip results.

Sample	Raw reads	Reads after trimming	Reads aligned to EquCab2.0	Reads assigned to target sites	Reads with variant called at site	Target loci genotyped	Genotype concordance
B1	9814	9622	8759	7258	7167	248	98.2
B28	7924	7786	6501	5328	5224	232	98.9
B33	22763	22243	20127	16872	16534	260	98.3
B36	15030	14741	13852	11658	11491	251	98.8
B39	154	153	137	107	105	5	100
E1	24727	24153	21027	18193	17845	257	98.4
E12	22386	21887	19863	16728	16423	258	98
E13	14751	14424	13156	10901	10783	253	99
E14	24111	23646	22491	19162	18916	260	98.3
E15	15245	14926	13887	11866	11655	253	98.4
E17	28675	28041	26447	22312	22018	260	97.9
E18	12847	12592	11901	9963	9808	247	99
E20	13773	13500	12130	10010	9848	252	98.6
E21	326	323	266	221	217	9	94.4

E25	24460	23944	22850	18651	18332	258	98.6
E26	14724	14420	13343	10906	10749	248	98.8
E28	22641	22193	20541	17443	17211	258	98.8
E3	26268	25799	24043	20708	20357	257	99
E33	38817	37960	35618	30554	30091	261	98.5
E34	676	664	583	503	495	41	97.6
E35	726	714	579	488	474	40	98.8
E38	482	478	426	346	340	17	100
E39	612	590	492	405	404	35	100
E7	9790	9525	7555	6333	6263	238	98.5
E8	12815	12594	11981	10192	10063	250	98.6
E9	6178	6047	5346	4407	4353	221	99.3
J104	26519	26002	24360	20846	20530	254	99.5
J108	36930	36162	32958	28394	27992	264	98.9
J37	8689	8508	7663	6455	6404	238	99
J51	7742	7575	6652	5563	5525	230	99
J52	11525	11299	10432	8516	8422	250	99.5
J61	4226	4125	3806	3247	3184	181	99.4
J69	19980	19511	17759	15188	14986	255	98.8

J91	21211	20860	20134	17449	17171	259	98.4
J92	8568	8375	7946	6669	6601	241	99.3
K102	15513	15197	12743	10980	10835	250	79.2
K109	22669	22241	19907	16879	16620	257	98.8
K115	16116	15793	12932	11131	10951	256	98.9
K122	2816	2751	2135	1870	1848	147	100
K30	24672	24167	23017	19702	19428	262	98.2
K46	22447	22015	20430	17329	17117	260	99.1
K6	17996	17558	15925	13310	13130	256	98.4
K68	32416	31770	28663	24538	24104	260	99.3
K80	29020	28515	27423	23411	22998	259	98.4
K96	10841	10634	9916	8383	8259	249	99.5
L32	23118	22688	20174	17135	16910	259	98.4
S104	23081	22557	21444	18006	17547	263	98
S97	27755	27194	25888	21662	21399	254	98.4
