# Supplementary material for: Targeted genome-wide SNP genotyping in feral horses using non-invasive fecal swabs
Source: Conserv Genet Resour. 2022 Mar 16;14(2):203–13. doi: 10.1007/s12686-022-01259-2 (PMC9162989; doi:10.1007/s12686-022-01259-2)
Supplement: Supplementary file 3 — Supplementary file3 (PDF 504 kb) [file 12686_2022_1259_MOESM3_ESM.pdf]

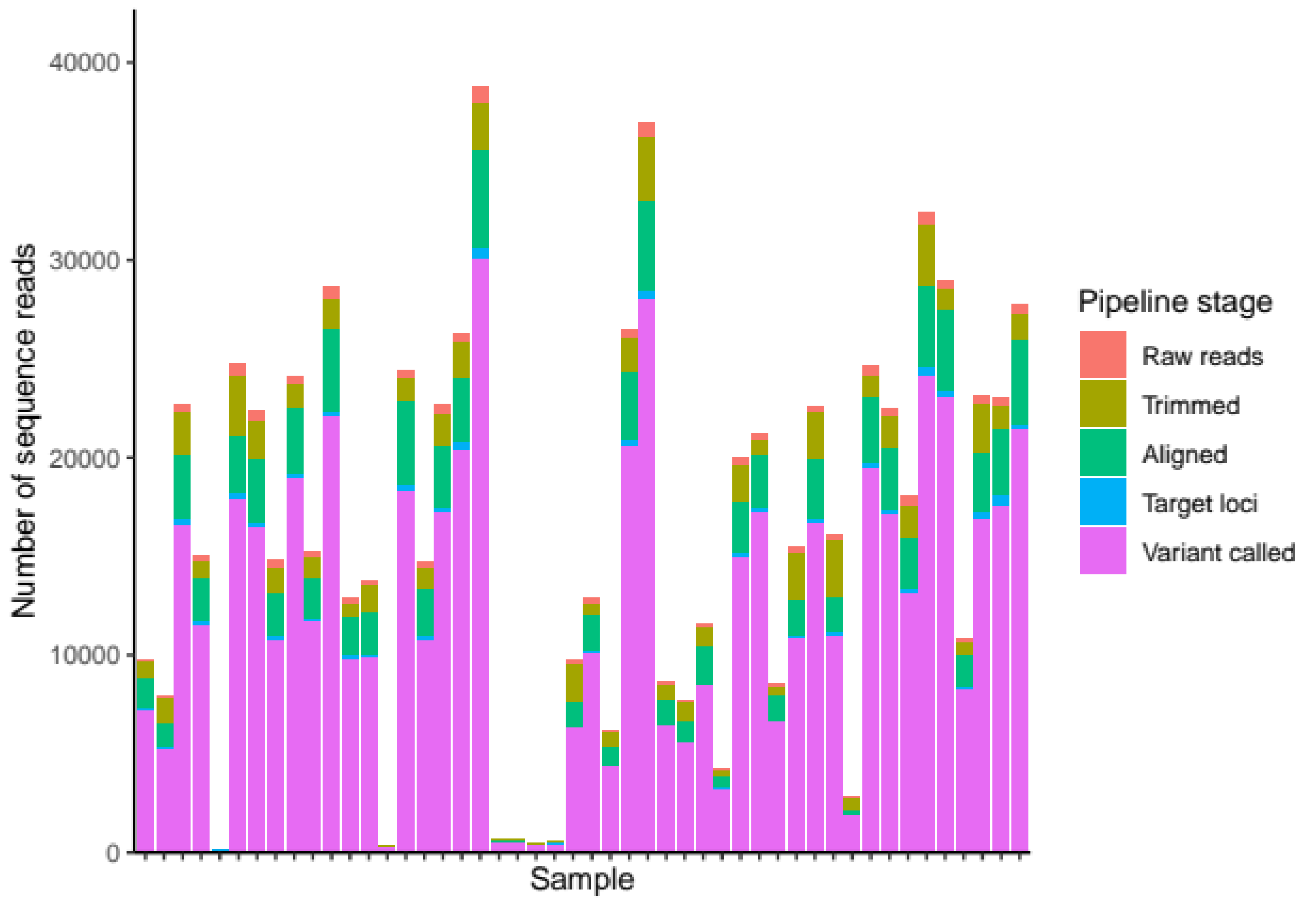

Online Resource 3. Summary of sequencing and bioinformatic processing for Allegro Targeted Genotyping of 279 targets for 48 fecal swab samples collected from feral horses on Sable Island, Nova Scotia, Canada. Presented are the number of raw reads generated and then, as a proportion of each bar representing a sample, the number of reads retained following processing steps: trimming, alignment to the EquCab2 reference genome, the number of reads assigned to a target site before variant calling, and the number of reads after variant calling.

Article: Targeted genome-wide SNP genotyping in feral horses using non-invasive fecal swabs

Journal: Conservation Genetics Resources

Authors: Stefan Gavriliuc[1], Salman Reza[2], Chanwoori Jeong[1], Fitsum Getachew[1], Philip D. McLoughlin[3], Jocelyn Poissant[1]*

Affiliations: [1] Department of Ecosystem and Public Health, University of Calgary, 3280 Hospital Drive, Calgary, AB T2N 4Z6, Canada

[2] Faculty of Veterinary Medicine, University of Calgary, Calgary, AB T2N 4Z6, Canada

[3] Department of Biology, University of Saskatchewan, 112 Science Place, Saskatoon, SK S7N 5E2, Canada

Corresponding author: Jocelyn Poissant (jocelyn.poissant@ucalgary.ca)
