# Supplementary material for: Targeted genome-wide SNP genotyping in feral horses using non-invasive fecal swabs
Source: Conserv Genet Resour. 2022 Mar 16;14(2):203–13. doi: 10.1007/s12686-022-01259-2 (PMC9162989; doi:10.1007/s12686-022-01259-2)
Supplement: Supplementary file 4 — Supplementary file4 (PDF 676 kb) [file 12686_2022_1259_MOESM4_ESM.pdf]

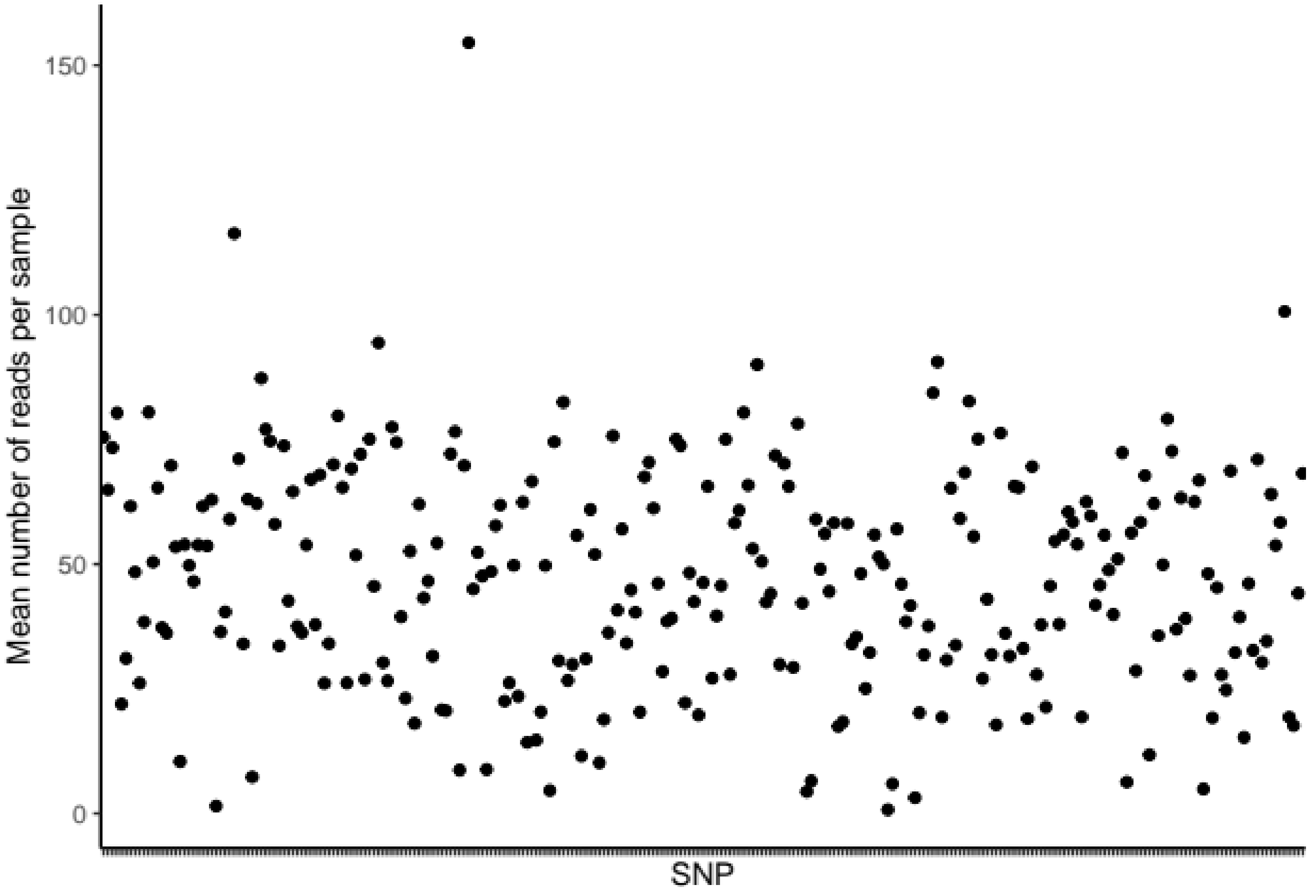


**Online Resource 4.** The mean number of reads at each targeted SNP loci across 48 samples. Genotypes were generated using Allegro Targeted Genotyping on 279 targets using 48 fecal swab samples collected from feral horses on Sable Island, Canada.

**Article:** Targeted genome-wide SNP genotyping in feral horses using non-invasive fecal swabs

**Journal:** Conservation Genetics Resources

**Authors:** Stefan Gavriliuc[1], Salman Reza[2], Chanwoori Jeong[1], Fitsum Getachew[1], Philip D. McLoughlin[3], Jocelyn Poissant[1]*

**Affiliations:** [1] Department of Ecosystem and Public Health, University of Calgary, 3280 Hospital Drive, Calgary, AB T2N 4Z6, Canada

[2] Faculty of Veterinary Medicine, University of Calgary, Calgary, AB T2N 4Z6, Canada

[3] Department of Biology, University of Saskatchewan, 112 Science Place, Saskatoon, SK S7N 5E2, Canada

**Corresponding author:** Jocelyn Poissant (jocelyn.poissant@ucalgary.ca)
